# Clinical outcomes following surgical management of deep infiltrating endometriosis

**DOI:** 10.1038/s41598-022-25751-9

**Published:** 2022-12-16

**Authors:** Perrine Leborne, Stephanie Huberlant, Florent Masia, Renaud de Tayrac, Vincent Letouzey, Lucie Allegre

**Affiliations:** 1 Department of Obstetrics and Gynecology, Nîmes University Hospital, Nîmes, France; 2 Department of Artificial Polymers, Max Mousseron Institute of Biomolecules, CNRS UMR 5247, University of Montpellier 1, Montpellier, France

**Keywords:** Pathology, Pregnancy outcome, Patient education

## Abstract

The main aim of the study was to evaluate severe post-operative complications following deep endometriosis surgery in a tertiary referral centre. This is a retrospective cohort study that included women who had surgery for deep infiltrating endometriosis between 1st January 2013 and 31st December 2019. Endometriosis was diagnosed based on clinical, imaging and histological parameters. We evaluated the rates of post-operative complications, potential risk factors for such complications and postoperative pregnancy rates. A total of 165 patients were included in the final analysis. The median follow-up was 63 (25–106) months. Thirty-seven patients (22.42%) had hysterectomy, 60 (36.81%) had ureterolysis and 44 (26.67%) had colorectal surgery. The overall and severe rates of post-operative complications were 16.20% (n = 23) and 2.42% (n = 4) respectively. Of the variables assessed, operative time and age were the only statistically significant risk factor for complications on multivariate analysis. Among women operated on for infertility, 34.5% (n = 20/58) got pregnant following surgery with 30% of these spontaneously. This study demonstrates acceptable overall and severe post-operative complications and pregnancy rates after deep endometriosis surgery. This information should help clinicians when counselling women to enable them making an informed choice about their management.

## Introduction

Endometriosis is defined as the presence of endometrial cells outside of the uterine cavity. There is wide variation in the reported prevalence of endometriosis. According to the French National Authority for Health (HAS) the prevalence varies between 2 and 74% in women suffering from chronic pelvic pain and it exceeds 33% in women presenting with acute pelvic pain^1^. The clinical presentation and impact of the disease are also variable and do not correlate to the extent of the endometriotic lesions^2^.

Surgical treatment is a management option when considering woman’s symptoms, the desire for pregnancy, age, severity of the condition and its site^1^. However, endometriosis surgery often entails a complex procedure necessitating extensive adhesiolysis with associated urological or gastrointestinal interventions. Studies that evaluated prognostic factors for operative complications in relation to endometriosis surgery suggest that it is associated with the depth of endometriotic lesions within the tissues, presence of recto-vaginal involvement, the patient’s age and previous surgical history^3,4^. It has been reported that the rate of major and minor complications following surgery for deep infiltrating endometriosis is around 3–4% and 10–15% respectively. The most commonly reported major complications are bowel related fistulae, particularly in patients with rectal or sigmoid colon involvement. Moreover, it is reported that 5–16% of women suffer with voiding dysfunction after such surgery^3–9^.

In view of these complications and the complexity of surgery for endometriosis, the French National Authority for Health recommends a multidisciplinary and specialised approach^1^. These recommendations were further supported by findings of a pilot expert centre study, which demonstrated improved clinical, patient reported and surgical outcomes. It also highlighted the potential research benefits of this centralised care^10^. These encouraging results were followed by the creation of several endometriosis expert centres for diagnostic, clinical care and applied research purposes^10^. Further support to these recommendations came from the reporting of initial results of these centres in a study that involved more than 490 patients, which revealed low complications rates for deep endometriosis surgery^5^. Nevertheless, severe cases of endometriosis were overrepresented in this study limiting the generalizability of its findings.

The French National Authority for Health and the French National college of gynaecologists and obstetricians (CNGOF) recommend that each endometriosis care centre would regularly report its endometriosis-related surgical outcomes and complications^11^. The main aim of this study was to review and report surgical complications rates following surgery for deep infiltrating endometriosis in our university affiliated hospital. We also wanted to explore potential risk factors for such complications and postoperative pregnancy rates.

## Materials and methods

This was a retrospective cohort study conducted in a tertiary referral university affiliated hospital. All experimental protocols were approved by the Institutional Review Board (IRB) of Nimes University Hospital n° 21.06.03.

We identified the study cohort by screening our electronic patient medical information system (PMSI) for patients undergoing surgery related to endometriosis. We used clinical codes corresponding to uterine, ovarian, Fallopian tube, pelvic peritoneum, vaginal, recto vaginal wall, bowel and cutaneous scar endometriosis between January 2013 and December 2019.

Informed consent was obtained from all subjects prior to any surgical intervention.

In our unit, the diagnosis of endometriosis is based on clinical symptoms (dysmenorrhea, dyspareunia or both), clinical examination and imaging involving MRI and/or ultrasound scans. MRI diagnosis depends on signal and morphologic abnormalities in line with previously published studies. Signal abnormalities are defined as hyperintense foci that correspond to hemorrhagic areas on T1-weighted MR images or small hyper intense cavities on T2-weighted MR images^12,13^. If rectal or sigmoid colon involvement is suspected, a colonoscopy combined with ultrasonography is performed to assess for the degree of gastrointestinal involvement complying with CNGOF recommendations^14^. If involvement of the anterior compartment was suspected, patients are often referred for cystoscopy to confirm or refute transmural bladder involvement. A pre or post-operative double J catheter is occasionally used if ureteric involvement is suspected. Women are considered for surgical excision of endometriosis if medical management failed to control their pain or to improve their fertility outcomes. The preferred surgical route is laparoscopic with the possibility of conversion to laparotomy in case of technical difficulties.

During the study period, the surgical procedures were performed by one of 5 different gynecological surgeons in association with a colorectal surgeon or a urologist in case of gastrointestinal or urinary tract involvement respectively. The gynecological surgeons comprised of 2 senior surgeons and 3 junior surgeons. However, junior surgeons operated under the senior surgeon’s supervision. All women were routinely given a post-operative follow-up appointment 6 weeks after surgery. Any excised tissue was sent for histological assessment. Only women with a confirmed histological diagnosis of endometriosis were included in our final analysis.

All methods were carried out in accordance with relevant guidelines and regulations.

Patients’ demographic, clinical, surgical and follow-up details were retrieved from their clinical hospital records. The primary endpoint of interest was the rate of severe post-operative complications. We used the Clavien Dindo classification^15–17^ to report the different complications and any complications classed as stage III or more were considered “severe”. Our secondary endpoints included the overall complications rate, length of hospital stay and re-admission rates. We also collected data on live birth rate following surgery, for the subgroup of women who underwent surgery for subfertility reasons.

Data are reported as the median and interquartile range (IQR), or number (n) and frequency, as appropriate. We used univariate analysis to identify potential risk factors for post-operative complications. All variables with a p < 0.2 and known risk factors based on previous studies (BMI and history of endometriosis surgery) were evaluated further using a multivariate logistic regression analysis. Results of this analysis are expressed as odds ratios (ORs) and 95% CI. A *p* value of < 0.05 was considered statistically significant. Statistical analysis was performed using R 2.9.2 (R Development Core Team (2009) R Foundation for Statistical Computing, Vienna, Austria).

## Results

### Study participants

**Figure 1:**
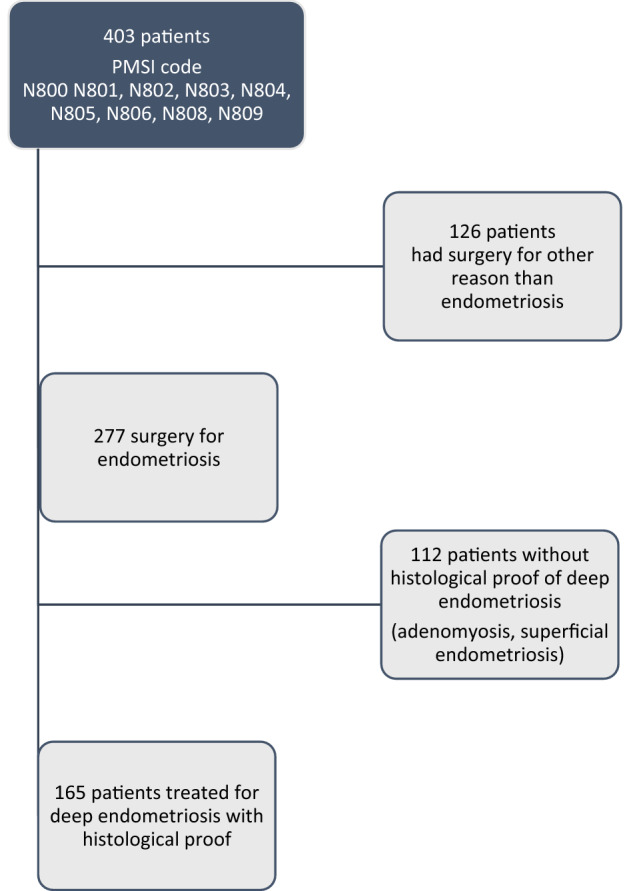
Flow chart of women screened and included into the study.

A total of 403 patients were identified from our initial screen of hospital electronic patient records. Of these, 126 women had surgery for, either another reason than endometriosis or had superficial endometriosis. The remaining 277 patients underwent surgery for endometriosis or adenomyosis. However, 112 women had no proof of the disease on histological examination or had adenomyosis without deep endometriosis leaving a total of 165 patients who fulfilled our inclusion criteria and contributed to the final analysis (Fig. 1).

The demographic details of our study cohort are presented in Table 1.

**Table 1:** Demographic characteristics of the study population.

Variable	Median (IQR) or n (%)	Missing data
Age	34.00 (11.00)	0 (0%)
BMI	23.00 (6.00)	0 (0%)
**ASA**		
Score 1	117 (70.91%)	0 (0%)
Score 2	45 (27.27%)	
Score ≥ 3	3 (1.82%)	
**Parity**		
Nulliparous	100 (60.61%)	2 (1.21%)
Parity = 1	34 (20.61%)	
Parity ≥ 2	29 (17.58%)	
History of endometriosis surgery	53 (31.93%)	0 (0%)
Number of previous surgeries	0.00 (1.00)	0 (0%)
Dysmenorrhea	119 (72.56%)	0 (0%)
Dyspareunia	87 (53.37%)	0 (0%)
Pain when defecating	20 (12.20%)	0 (0%)

### Operative details

The main indications for surgery were pain, infertility and both pain and infertility in 104 (63%), 33 (20%) and 24 (14.6%) women respectively. Four patients (2.4%) had surgery for dysmenorrhea and heavy menstrual bleeding. The median operative time was 126 (IQR = 110.75) min and the median hospital stay was 3 (IQR = 3) days. Surgery was performed laparoscopically in 160 cases (96.97%); one of these was converted to laparotomy because of the need for ureteric implantation. Two patients had combined laparoscopic and vaginal procedures because of vaginal nodules, one had robotic-assisted surgery and 2 had a laparotomy because of previous complex surgical histories.

Hysterectomy was performed in 37 patients (22.42%), Bowel surgery was required in 44 cases (26.67%), of these 8 (18.18%) had segment resection, 2 (4.55%) had a discoid resection and 34 (77.27%) had bowel shaving of endometriotic deposits. Excision of the nodules infiltrating the rectovaginal septum was performed laparoscopically controlled by frequent vaginal assessments. Six patients (13.63%) had a temporary protective ileostomy following their resection anastomosis. Of these, 5 were performed immediately and one as a delayed procedure when the patient was re-operated on. Sixty patients (36.81%) had ureterolysis and 5 (3.03%) had vesical resection. All surgical procedures are presented in Table 2.

**Table 2:** Surgical procedures and pelvic structures involvement.

Variable	n (%)
**Gynaecological structures involvement**	
Excision of nodules on uterosacral ligament	48 (29.09%)
Excision of nodules on the torus uterinum	22 (13.41%)
Hysterectomy	37 (22.42%)
Salpingectomy	58 (35.15%)
Ovarian cystectomy	69 (41.82%)
**Urological involvement**	
Ureterolysis	60 (36.81%)
Ureteral reimplantation	3 (1.82%)
Excision of nodules in vesico uterine space	10 (6.06%)
Bladder resection	5 (3.03%)
**Bowel involvement**	
Rectal surgery	44 (26.67%)
Segment resection	8 (18.18%)
Discoid resection	2 (4.55%)
Shaving	34 (77.27%)
Ileocaecal resection	1 (0.61%)
Appendicectomy	4 (2.42%)
Protective stoma	6 (13.63%)

### Outcomes of interest

Our study cohort had a median follow-up duration of 63 (IQR = 81) months. Among the 165 patients operated on for deep invasive endometriosis, 20.6% (n = 34) had recurrence of their pain after a median time of 12 (IQR = 83) months from surgery. For which, a total of 23 women (13.9%) had to have an additional surgical procedure after a median period of 22 (IQR = 82) months from their initial surgery.

Based on the Clavien Dindo classification, among the 165 patients, 2.42% (n = 4) had severe complications, where 3 (1.82%) women had grade IIIB and one (0.61%) had grade IVA complications. Two of these complications occurred at the beginning and 2 at the end of the study period. With regards to the IIIB complications, 2 patients had to be re-operated on following a hysterectomy, one had vaginal vault dehiscence and the other had excessive vaginal bleeding 15 days after her primary surgery. The third patient presented with fever and investigations suggesting acute inflammation 5 days after bilateral ureterolysis and rectal shaving of endometriotic nodules. The patient was diagnosed with a ureteric fistula. The fistula healed spontaneously after the insertion of a left ureteral stent.

The patient presenting with a grade IVA complication had a hysterectomy with right adnexectomy and recto-sigmoid junction resection. She presented with an anastomotic fistula 3 days after surgery requiring a re-operation for peritoneal lavage, drainage and an ileostomy. Her postoperative recovery was complicated by a septic shock and renal dysfunction. The patient was discharged 44 days after her repeat surgery with fully recovered renal function.

Four patients (2.42%) had to be re-admitted after a median period of 15 days (IQR = 20). Two of these were the grade IIIB complication patients described above. The third was re-admitted for suspected intestinal obstruction, which resolved spontaneously. While, the fourth patient was admitted for a cystography prior to removing her urinary catheter inserted for an intraoperative bladder injury.

The overall rate of complications was 16.20% (n = 23). The most frequent complications were urinary tract infections (6.06%, n = 10) and unexplained fever (3.64%, n = 6). Pyelonephritis and an abdominal wall abscess occurred in 2 patients (1.21%) each. Post-operative voiding dysfunction occurred in 1.82% of cases (n = 3). Overall, the median time for onset of complications was within 6 (IQR: 11.75; range: 1–90) days post-operative. The full list of complications is presented in Table 3.

**Table 3:** Complications.

Variable	n (%)
Post-operative complication*	23 (16.20%)
**Clavien Dindo classification**	
I	4 (2.42%)
Abdominal wall abscess	2 (1.22%)
Intestinal obstruction	1 (0.61%)
Voiding dysfunction	3 (1.82%)
II	15 (9.09%)
Unexplained fever	6 (3.64%)
Urinary tract infection	10 (6.06%)
Pyelonephritis	2 (1.21%)
III	3 (1.82%)
Vaginal vault dehiscence	2 (1.21%)
Ureteral fistula	1 (0.61%)
IV	1 (0.61%)
Bowel anastomotic leakage	1 (0.61%)

*Some patients had more than one complication.

Among women operated on for infertility (n = 58), 34.5% (20/58) had successful pregnancies after surgery, of these, 70% and 30% had medically assisted and spontaneous conceptions respectively. The median duration from surgery to conception was 9 (IQR = 35) months.

On univariate analysis, rectal surgery (OR 4.28 [1.98–9.25]; p < 0.001) and operative time (OR 1.01 [95% CI 1.01–1.02]; p < 0.001) were significantly associated with post-operative complications. However, on multivariate analysis, only the operative time remained significant (aOR 1.01 [95% CI 1.00–1.01]; p = 0.009). Additionally, age was also identified as an independent risk factor for post-operative complications (aOR 1.08 [1.02, 1.14]; p = 0.005) (Table 4).

**Table 4:** Logistic regression predicting post-operative complication: univariate and multivariate analysis:

	Univariate analysis		Multivariate analysis	
	OR (95% CI)	p value	Adjusted OR (95%CI)	p value
Rectal surgery	4.28 (1.98–9.25)	< 0.001	2.84 (0.89–9.12)	0.079
BMI	0.99 (0.93–1.05)	0.706	0.96 (0.89–1.04)	0.235
Age	1.04 (1.00–1.09)	0.077	1.08 (1.02–1.14)	0.005
Previous endometriosis surgery	1.19 (0.56–2.56)	0.65	0.83 (0.36–1.94)	0.665
Operative time	1.01 (1.01–1.02)	< 0.001	1.01 (1.00–1.01)	0.009
Colpotomy	1.59 (0.17–14.63)	0.682		

## Discussion

This study provides an overview of several clinical outcomes following surgical management of deep infiltrating endometriosis in a tertiary hospital in France. We report a rate of severe post-operative complications of 2.42% which is consistent with previous studies^7–9^. Our findings are also in agreement with other studies, which evaluated rates of complications and their risk factors in endometriosis surgery^18,19^. Lermann et al.^8^ described a population of 134 patients who had surgical management for deep endometriosis and reported 3.7% and 12.7% rates of severe and minor complications respectively. These rates are comparable to our results, although, they excluded patients who required bowel resection. Of relevance, two of the patients in our study who presented with severe complications had extensive endometriosis involving their bowels requiring shaving or resection.

The main complication after surgery in our study was urinary tract infection, which occurred in 6.06% of our cohort. Urinary tract infections might be explained by the fact that patients had a urinary catheter during and following surgery to mitigate the risk of post-operative voiding dysfunction. It has been shown that bacteriuria occurred more often with increased length of catheterisation^20^. However the risk of infection secondary to catheter insertion should be weighed against its benefits. Indeed, only 3 women included in our study had voiding dysfunction (1.82%) after surgery. In a comprehensive literature review, Campin et al. reported varying risk of post-operative voiding dysfunction ranging from 0.8 to 30%^21^. Furthermore, this rate has been more recently reported to be as high as 50% by others^22^.

In our center, ureteral stent was placed before surgery only in if there is uretero hydronephrosis. Moreover, systematic placement of a ureteral stent does not seem to reduce overall perioperative complication rate and could be associated with a longer duration of hospitalization and a higher rate of urinary tract infections^23^.

The rate of temporary protective ileostomy in our study was 13.63%, which is comparable to previous reports. Indeed, in a national snapshot of the surgical management of deep infiltrating endometriosis of the rectum and colon in France in 2015, Roman et al. reported a protective stoma rate of 19%^24^. More recently, it was demonstrated that a protective stoma did not prevent the occurrence of a recto-vaginal fistula^25^. This is consistent with our results where our restrictive protective ileostomy policy was still associated with a low rate of serious complications. To reduce the rate of complications after colorectal procedures, conservative surgery, as rectal shaving and discoid excision, have been proposed. Roman et al., on behalf of the FRIENDS group, conducted a large national retrospective study including 1135 patients undergoing surgery for deep endometriosis of the rectum and colon. They showed significantly higher rate of recto-vaginal fistula following rectal resection compared to conservative treatment^24^. Findings from our centre also support this notion. Moreover, conservative surgery could improve functional results and reduce voiding dysfunction or LARS (low anastomotic resection syndrome)^26^. However, due to the retrospective nature of our study, we are not able to report de novo bowel or urinary complaints. It would be interesting to explore this issue further in future studies by assessing urinary and bowel functions pre and post-operatively^27^.

Nowadays, some studies highlight the potential benefit of relatively new techniques for the evaluation of anastomotic vascularization, like the indocyanine green. This test was not routinely used at the time of our study cohort. However, this technique could be useful to reduce post operative complication after bowel endometriosis surgery^28,29^.

Among the severe complications in our study, 2 occurred at the beginning and 2 at the end of our studied period. However, with advances in surgical techniques, specialisation and centralisation of care the number of women who had surgical management of endometriosis have increased over the years. In a large multicenter retrospective study, Bendifallah et al.^30^ demonstrated that the rate of surgical complications when managing deep colorectal endometriosis was independently inversely linked to the annual number of procedures performed by centre and surgeon. In their study, the authors suggested that the optimal number of procedures required per surgeon per year to be between 7 and 13 operations. In our centre, the mean number of colorectal procedure performed per year was 7.

It has been reported that the main surgical risk factors during deep endometriosis surgery are the opening of the vagina, a low colorectal anastomosis and resection of endometriosis in relation to the urinary bladder and ureters^18,31,32^. In our study, colpotomy was not identified as a risk factor for post-operative complications. Moreover, although rectal surgery was a significant factor on univariate analysis, it was not on multivariate analysis. However, it is possible that due to the small number of patients who had rectal surgery in our cohort, our study did not have enough power to demonstrate such association. Diabetes and obesity have been identified to be risk factors of wound and anastomosis healing^33–35^. The two patients with the most severe complications in our study did not have diabetes, but had body mass indices of 32 and 37. Nevertheless, body mass index (BMI) was not identified as a risk factor in our cohort, which could be because our study population were mostly not obese with a median BMI of 23 (IQR = 6). This may also be related to a lack of power in our study to demonstrate such a difference or due to the predominant use of laparoscopy, which tends to be associated with fewer complications related to high BMI compared to laparotomy^36^.

Operative time was identified as a risk factor for post-operative complications in our study. Arguably, complex surgical procedures with extensive adhesiolysis and bowel resections require longer operative time; nonetheless, this was demonstrated as an independent factor on multivariate analysis. Poupon et al. reported that the ENZIAN score is a good predictor of complications^3^. Other authors have studied the correlation between MRI and the ENZIAN score and showed good correlation between both^37,38^. We were not able to assess the predictive effect of this score on surgical complication rates in our patients because it was not used in our unit during the study period.

This study has several strengths, which include the use of our comprehensive electronic patient record and clinical coding system to identify our study cohort minimising the risk of selection bias. Additionally, we mitigated the risk of contaminating our analysis by having clear and strict inclusion criteria. This has ensured that our findings are relevant to the group of women considered at higher risk for surgical management of their endometriosis. Therefore, we believe that our findings will help provide women with realistic information to enable them make an informed choice about their care. Finally, the use of an internationally recognised system to categorise post-operative complications adds to the external validity of our findings. However, we also recognise the limitations posed by the retrospective design of our study. We are aware that the heterogeneity of surgery could represent a source of bias. However, the aim of this study was to report the post operative complication rate in routine practice. Specific complications of severe endometriosis surgery are well reported by expert center publications. Moreover, our study would not have had the power to produce meaningful results in specific surgery. Furthermore, the low rate of occurrence of severe complications could be perceived as a limitation. Nevertheless, our identified rates concur with other studies involving independent cohorts.

In conclusion, the rate of severe post-operative complications after deep endometriosis surgery in a specialised centre is low. Our study demonstrated that operative time and age were independent risk factors for postoperative complications. This study adds evidence to the importance of multidisciplinarity and centralisation of care in the surgical management of deep infiltrating endometriosis. This information should help clinicians when counselling women to enable them make an informed choice about their management.

## Data Availability

The datasets used and/or analyzed during the current study are available from the corresponding author on reasonable request.
